# γδ T-cells in HIV infection

**DOI:** 10.1186/1742-4690-9-S2-P200

**Published:** 2012-09-13

**Authors:** NG Holt, J Johnson, S Wilton, E Byrne, A Piechocka-Trocha, BD Walker, D Kwon

**Affiliations:** 1 Ragon Institute of MIT, MGH and Harvard, Boston, MA, USA; 2 Howard Hughes Medical Institute, Chevy Chase, MD, USA

## Background

γδ T-cells represent a first line of defense against pathogens in the mucosa. Despite their prevalence in gut associated lymphoid tissue (GALT), little is known about their role in HIV infection. We hypothesize that γδ T-cells are stimulated by viral antigen and demonstrate anti-HIV activity, comprising a critical component of the mucosal response to HIV.

## Methods

To assess the role of γδ T-cells, we analyzed peripheral blood and GALT samples from HIV(-) and HIV(+) patients, including elite controllers. γδ T-cells were isolated and assessed in viral inhibition and CD4+ killing assays. The cellular pathway associated with cell killing was also evaluated. An HIV antigen screen was used to stimulate sorted γδ T-cells. Nanostring analysis was used to measure mRNA. High-throughput TCR sequencing was performed in peripheral and mucosal tissue.

## Results

The mucosal subtype, Vδ1, exists at higher percentages in HIV(+) peripheral blood, particularly elite controllers (17.1±4.0), relative to HIV(-) subjects (0.3±0.2) (p=0.0001). A 100-fold increase of the Vδ1 subtype was detected in the ileum of HIV controllers. Vδ1 cells in the GALT of HIV(-) patients, unlike those in the periphery, directly kill up to 80%±20% of HIV+CD4+ T-cells in culture and inhibiting virus production by 3 logs. These antiviral effects are expanded to the periphery in the setting of elite control. γδ T-cell mediated killing is correlated to perforin expression (R=0.8088). Nef-specific responses in Vδ1 cells were observed in patients with lower viral loads and higher CD4+ count indicating that antiviral effects may be mediated by an HIV-specific response (p=0.01).

## Conclusion

γδ T-cells play a key role in the response to HIV infection. HIV specific γδ T-cells are expanded from mucosal tissue to the periphery where they exert anti-viral effects. Further study may suggest ways to harness this unique subset to stimulate both innate and acquired immunity in response to HIV.

